# Fraxetin Inhibits the Proliferation and Metastasis of Glioma Cells by Inactivating JAK2/STAT3 Signaling

**DOI:** 10.1155/2021/5540139

**Published:** 2021-04-16

**Authors:** Liangchen Qu, Pan Lin, Minjie Lin, Shumin Ye, Percy David Papa Akuetteh, Youyou Zhu

**Affiliations:** ^1^Taizhou Hospital of Zhejiang Province Affiliated to Wenzhou Medical University, Taizhou, Zhejiang, China; ^2^Linhai Center for Disease Control and Prevention, Taizhou, Zhejiang, China; ^3^Key Laboratory of Diagnosis and Treatment of Severe Hepato-Pancreatic Diseases of Zhejiang Province, Zhejiang Provincial Top Key Discipline in Surgery, Wenzhou Medical University First Affiliated Hospital, Wenzhou, Zhejiang, China

## Abstract

Glioma is the most common brain tumor and is characterized by high mortality rates, high recurrence rates, and short survival time. Migration and invasion are the basic features of gliomas. Thus, inhibition of migration and invasion may be beneficial for the treatment of patients with glioma. Due to its antitumor activity and chemical reactivity, fraxetin has attracted extensive interest and has been proven to be an effective antitumor agent in various cancer types. However, currently, the potential effects of fraxetin on glioma have not been investigated. Here, we demonstrate that fraxetin can inhibit the proliferation, invasion, and migration of glioma and induce apoptosis of glioma cells *in vitro* and *in vivo*. Therefore, these findings establish fraxetin as a drug candidate for glioma treatment. Furthermore, fraxetin was able to effectively inhibit the JAK2/STAT3 signaling in glioma. In summary, our results show that fraxetin inhibits proliferation, invasion, and migration of glioma by inhibiting JAK2/STAT3 signaling and inducing apoptosis of glioma cells. The present study provides a solid basis for the development of new glioma therapies.

## 1. Introduction

Glioma, the most common primary malignant brain tumor, is characterized by high mortality rates, high recurrence rates, and short survival time of patients [1, 2]. The median survival time for patients diagnosed with glioma is less than 16 months, even though surgical resection combined with chemotherapy and radiotherapy is widely used to treat glioma [3, 4]. Thus, there is an urgent need to find new strategies for glioma treatment.

Activation of the Janus kinase/signal transducer and activator of transcription (JAK/STAT) signaling cascade is correlated with diverse cancer types, including pancreatic cancer, hepatocellular carcinoma, and glioma [5, 6]. This pathway is involved in various cellular processes, such as cellular differentiation, proliferation, migration, and apoptosis in mammals [7]. Thus, treatment targeting the JAK/STAT signaling pathway may be beneficial for cancer patients, not only through inhibiting the growth of tumor but also by inducing apoptosis of cancer cells [8]. Fraxetin is a plant-derived coumarin, mainly isolated from *Fraxinus bungeana*, reported to have potent anti-inflammatory, antibacterial, and neuroprotective effects with low toxicity [9–11]. Sánchez-Reus et al. found fraxetin might prevent the apoptotic death of dopaminergic cells induced by rotenone and mediated by oxidative stress [10]. In recent years, studies have shown that fraxetin can inhibit tumor growth and metastasis in various types of cancer, including lung cancer, breast cancer, and osteosarcoma [12]. Importantly, fraxetin can inhibit the proliferation of non-small-cell lung cancer cells by preventing activation of STAT3 [13]. Considering the observed overactivation of JAK2/STAT3 in glioma [14], we speculated that fraxetin might have an inhibitory effect on glioma development via suppressing JAK2/STAT3 activity.

In this study, we aimed to identify the effects of fraxetin on glioma *in vitro* and *in vivo* and further elucidated its potential molecular mechanisms. In addition, we evaluated the activity of JAK2/STAT3 signaling in glioma. Our results suggested that fraxetin not only inhibits the proliferation, invasion, and migration of glioma but also induces glioma cells apoptosis by suppressing activation of JAK2/STAT3 signaling pathway. Thus, fraxetin may be a promising candidate for the treatment of glioma.

## 2. Materials and Methods

### 2.1. Drugs

Fraxetin (CAS: 574-84-5, purity of 99.77%) was purchased from MedChemExpress (MCE, Shanghai, China) and dissolved in DMSO at a concentration of 100 mM.

### 2.2. Cell Line and Cell Culture

The human glioma cell line U251 and U87 and normal cell line BV2 were obtained from the Shanghai Institute of Biological Sciences and Cell Resource Center (Shanghai), China. All kinds of cells were cultured in Dulbecco's Modified Eagle Medium (DMEM, Invitrogen, California, USA) supplemented with 10% fetal bovine serum (FBS, Invitrogen), 100 *μ*g/ml streptomycin, and 100 U/ml penicillin. The U251 cells were maintained in a humidified atmosphere of 5% CO_2_ at 37°C and passaged every two to three days.

### 2.3. CCK-8 Assay

Cell Counting Kit-8 (CCK-8) assay was carried out to determine the viability of U251 cells. The cells were plated in 96-well plates (2 × 10^3^ cells/well) in four replicates. The cells were then treated with fraxetin and after that adhered and cultured at 37°C for another 24 h. Finally, 10 *μ*l CCK-8 reagent was added to each well and then incubated for two to three hours, and then, the absorbance was measured at 450 nm [15]. The experiment was repeated at least three times independently.

### 2.4. Flow Cytometry Analysis

Flow cytometry analysis was used to detect the apoptosis of U251 cells treated with fraxetin. Briefly, the cells were plated in 6-well plates at a density of 3 × 10^4^ cells/well. After the cells were adherent, they were treated with different concentrations of fraxetin (0 *μ*M, 100 *μ*M, and 200 *μ*M) and cultured at 37°C for another 24 h. In order to analyze the apoptosis, cells were resuspended in 200 *μ*l binding buffer containing 10 *μ*l Annexin V-FITC and incubated for 25 min in the dark at room temperature. Next, 300 *μ*l binding buffer was added and mixed with 5 *μ*l propidium iodide (PI) for another 5 min. Finally, the rate of cell apoptosis was measured with a flow cytometer (BD FACSCalibur, USA) [16]. The experiment was repeated at least three times independently.

### 2.5. Immunofluorescence Staining

Cells were cultured in a 6-well plate and treated with concentrations of fraxetin for 24 h. Then, the cells were washed two times with PBS and fixed with 4% paraformaldehyde at room temperature for 30 min. Next, the cells were permeated in 0.1% Triton X-100 for 15 minutes and then washed five times with PBS, after which they were blocked with 10% FBS for 60 minutes. The cells were incubated overnight at 4°C with p-STAT3 (1 : 200, Abcam, ab76135), which was used as the primary antibody. After washing with PBS for five times, the cells were then incubated with secondary antibody at 37°C for 1 h. To stain the nucleus, DAPI was added after washing the cells with PBS five times. Finally, images were captured under a fluorescence microscope [17]. The experiment was repeated at least three times independently.

### 2.6. Real-Time Cellular Analysis (RTCA)

For proliferation assay, U251 cells were seeded into E16-plate (ACEA Biosciences, USA) at a density of 2.5 × 10^4^ cells/well. After the cells were adhered, they were treated with fraxetin (0 *μ*M, 100 *μ*M, and 200 *μ*M), and then, an unlabeled real-time cell analysis (RTCA) system was used to record the growth index of U251 cells [18]. The experiment was repeated at least three times independently.

### 2.7. Transwell Invasion Assay

Transwell invasion assay was performed to detect the invasion ability of U251 cells. The chambers were coated with Matrigel ®, and then, U251 cells were diluted in 200 *μ*l serum-free DMEM containing fraxetin and then inoculated in the upper chamber at a density of 1.5 × 10^5^ cells/well. The lower chamber was filled with 500 *μ*l DMEM containing 10% FBS. The cells were incubated for 24 h. Q-TIP was used to wipe the cells on the upper surface of the membrane, and then the invaded cells on the lower surface were fixed with formaldehyde for 30 min and washed with PBS two times. Next, the cells were stained with 0.5% crystal violet (Sigma) for 15–25 min. Finally, the images were captured under a microscope [19]. The experiment was repeated at least three times independently.

### 2.8. Colony Formation Assay

The U251 cells were plated in 6-well plates at a density of 500–600 cells/well. After the cells were adhered, they were treated with fraxetin (0 *μ*M, 100 *μ*M, and 200 *μ*M) and cultured at 37°C for 24 h. Next, the medium was replaced with a fresh one and cultured for another 10–14 days. The colonies were fixed with formaldehyde for 30 min and then washed twice with PBS. Finally, the cells were stained with crystal violet, and the cell colonies were counted [20]. The experiment was repeated at least three times independently.

### 2.9. Wound Healing Assay

In order to detect the migration ability of U251 cells, wound healing assay was performed. Briefly, the cells were seeded into a 6-well plate at a density of 5 × 10^4^ cells/well and cultured at 37°C for 24 hours. Next, a linear scratch was made on the plate using the crystal pipette tip. The cells were washed with PBS before the addition of new culture media containing fraxetin (0 *μ*M, 100 *μ*M, and 200 *μ*M). After 24 hours, the mages of our results were then captured under an inverted microscope [21]. The experiment was repeated at least three times independently.

### 2.10. Western Blot Analysis

The U251 cells were treated with fraxetin (0 *μ*M, 100 *μ*M, and 200 *μ*M) and cultured in 6-well plate for 24 h. The total proteins of U251 cells were collected, and concentrations of protein were measured by BCA (Beyotime Biotechnology). Next, 50 *μ*g of proteins was separated by SDS-PAGE and transferred to PVDF membrane (Solarbio, Beijing, China). The membrane was then blocked with 5% non-fat milk for 1.5–2 h and incubated with primary antibodies overnight at 4°C. The primary antibodies used were as follows: Ki67 antibody (1 : 1000, ab92742, Abcam), PARP antibody (1 : 1000, ab191217, Abcam), cleaved PARP antibody (1 : 1000, ab32064, Abcam), procaspase 8 antibody (1 : 1000, ab108333, Abcam), cleaved caspase 8 antibody (1 : 1000, 13423-1-AP, Proteintech), procaspase 3 antibody (1 : 1000, ab32150, Abcam), cleaved caspase 3 antibody (1 : 1000, 19677-1-AP, Proteintech), JAK2 antibody (1 : 1000, ab108596, Abcam), p-JAK2 antibody (1 : 1000, ab32101, Abcam), STAT3 antibody (1 : 1000, ab68153, Abcam), p-STAT3 antibody (1 : 1000, ab76315, Abcam), MMP-2 antibody (1 : 1000, ab92536, Abcam), and MMP-9 antibody (1 : 1000, ab76003, Abcam). The membrane was washed three times in TBST and incubated with anti-rabbit secondary antibody (1 : 5000, Proteintech) for 1 h. For internal reference, GADPH (1 : 1000, MB9231, Bioworld) was used as the internal reference. Finally, the immunoreactive bands were washed in TBST five times and visualized using chemiluminescence detection on autoradiographic film. The experiment was repeated at least three times independently.

### 2.11. Animal Experiments

Eight-week-old female nude mice (BALB/c) were obtained from the Experimental Animal Center of Wenzhou Medical University (Wenzhou, China). The right lower extremity of nude mice was injected subcutaneously with 4 × 10^6^ U251 cells. The experimental mice (*n* = 5) received intragastric administration of fraxetin (30 mg/kg) every three days for 30 days, whereas control mice (*n* = 5) were administered with saline. The length and width of tumor were measured every four days, and tumor volumes were calculated according to V = (length × width^2^)/2. At the end of the experiment, the mice were killed by a lethal dose of carbon dioxide to check for tumor formation, and the air displacement was 20%/min. Animal experiments, including animal euthanasia, were carried out in accordance with all regulatory institutional guidelines for animal welfare (National Institutes of Health Publications, NIH Publications no. 80–23).

### 2.12. Statistical Analysis

The results were presented as the mean ± standard deviations. The statistics were performed using GraphPad Prism (version 6.02) software. We performed a dual-sided Student's *t*-test to analyze the differences between two groups, and one-way ANOVA was used for the comparison of more than two groups. *P* < 0.05 was considered statistically significant. Pairwise group comparisons were conducted using Tukey's test as a post-hoc test following ANOVA. All the experiments were repeated at least three times.

## 3. Results

### 3.1. Fraxetin Inhibits the Proliferation of Glioma by Downregulating Ki67 Expression

**Figure 1:**
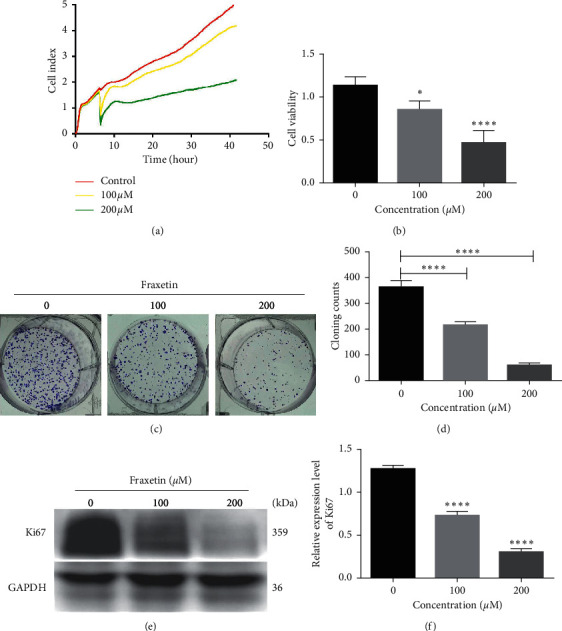
Fraxetin inhibits the proliferation of the U251 cells. (a) RTCA assay. Cells were treated with fraxetin (0 *μ*M, 100 *μ*M, and 200 *μ*M), and cell index was automatically recorded every 15 min. (b) CCK-8 assay. Cells were treated with fraxetin (0 *μ*M, 100 *μ*M, and 200 *μ*M) for 24 h. ((c) and (d)) Colony formation assay. Cells were treated with fraxetin (0 *μ*M, 100 *μ*M, and 200 *μ*M) for 14 days. ((e) and (f)) Western blot analysis of Ki67 protein in U251 cells treated with fraxetin (0 *μ*M, 100 *μ*M, and 200 *μ*M). ^*∗*^*P* < 0.05; ^*∗∗∗∗*^*P* < 0.0001.

Unlabeled real-time cell analysis (RTCA) and CCK-8 assay were carried out to investigate the effect of fraxetin on the proliferation of glioma cells. The results of RTCA showed that the viability of U251 cells gradually decreased with increasing the concentration of fraxetin (0 *μ*M, 100 *μ*M, and 200 *μ*M) (Figure 1(a)). Similarly, the CCK-8 results of U251 cells further proved that fraxetin had a stronger antiproliferative activity in glioma (Figure 1(b)). Similar results can also be observed in U87 cells (Figure S1B). Besides, we found that fraxetin had a low toxicity on normal cells BV2 (Figure S1A). In addition, colony formation assay revealed that fraxetin reduced the number of colony formation in U251 cells (Figures 1(c) and 1(d)).

A previous study has shown that Ki67 is a cellular marker of proliferation. We found that fraxetin significantly decreased the expression of Ki67 protein (Figures 1(e) and 1(f)). Thus, our results suggest that fraxetin has antiproliferation activities on glioma in a dose-dependent manner.

### 3.2. Fraxetin Induces Apoptosis in Glioma Cells through Mitochondrial-Dependent Pathway

**Figure 2:**
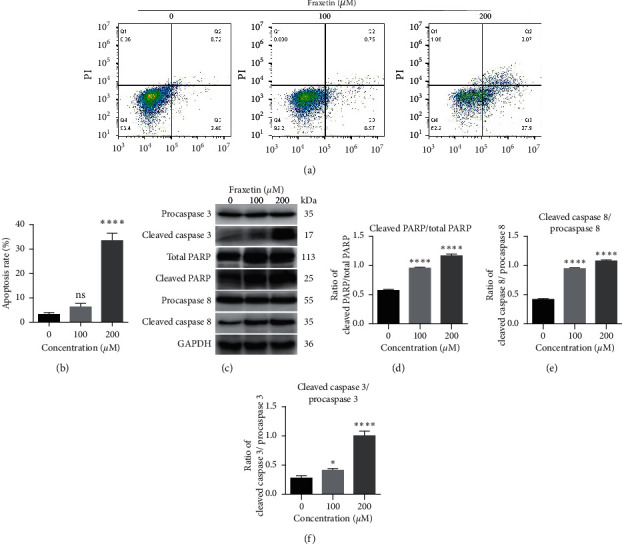
Fraxetin induces the apoptosis of U251 cells. ((a) and (b)) Annexin V-FITC/PI staining and flow cytometric analysis. Cells were treated with fraxetin (0 *μ*M, 100 *μ*M, and 200 *μ*M) for 24 h, and apoptosis rate were measured. ((c), (d), (e), and (f)) Western blot analysis of apoptosis-related proteins in U251 cells treated with fraxetin (0 *μ*M, 100 *μ*M, and 200 *μ*M). ^*∗*^*P* < 0.05; ^*∗∗∗∗*^*P* < 0.0001.

Considering the antiproliferation activity of fraxetin in glioma, to determine whether fraxetin can induce cellular apoptosis, the apoptosis of U251 cells was examined by flow cytometry. Our results revealed that fraxetin could increase the ratio of apoptotic cells in a concentration-dependent manner (Figures 2(a) and 2(b)). Next, we detected the expression levels of apoptosis-related proteins. Fraxetin treatment increased the ratio of cleaved caspase-8/procaspase 8, cleaved caspase 3/procaspase 3, and cleaved PARP/full length PARP (Figure 2(c)–2(f)). These findings revealed that fraxetin induces the apoptosis of U251 cells by mitochondrial-dependent pathway.

### 3.3. Fraxetin Inhibits Invasion and Migration of Glioma Cells

**Figure 3:**
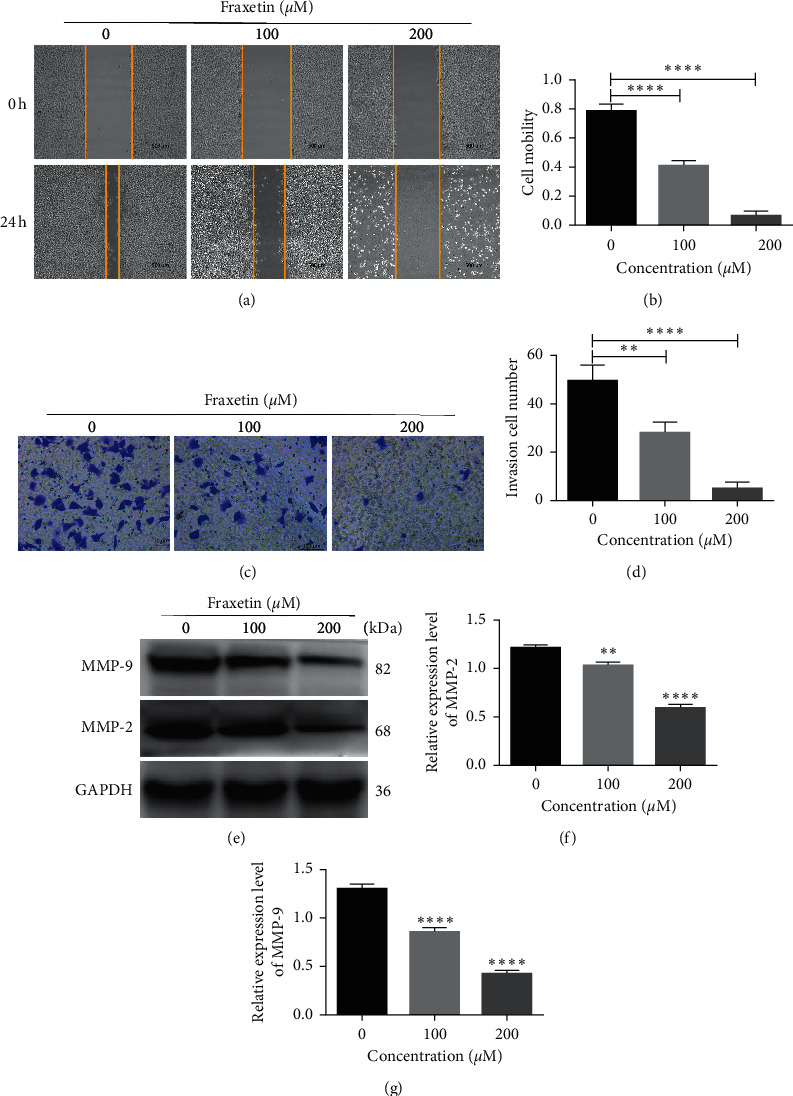
Fraxetin inhibits the invasion and migration of U251 cells. ((a) and (b)) Wound healing assay. Cells were treated with fraxetin (0 *μ*M, 100 *μ*M, and 200 *μ*M) for 24 h. ((c) and (d)) Transwell invasive assay. Cells were treated with fraxetin (0 *μ*M, 100 *μ*M, and 200 *μ*M) for 24 h. ((e), (f), and (g)) Western blot analysis of metastasis-related proteins in U251 cells treated with fraxetin (0 *μ*M, 100 *μ*M, and 200 *μ*M). ^*∗∗*^*P* < 0.01; ^*∗∗∗∗*^*P* < 0.0001.

Transwell invasion assay and wound healing assay were performed to evaluate the effects of fraxetin on invasion and migration of glioma cells. As shown in Figures 3(a) and 3(b), the migration ability of U251 cells decreased gradually with an increase in the concentration of fraxetin. In addition, the invasion ability of the cells was significantly inhibited by fraxetin (Figures 3(c) and 3(d)). Similar results can also be observed in U87 cells (Figures S1C and S1D). Furthermore, the study showed that fraxetin treatment decreased the expression of MMP-2 and MMP-9 protein (Figures 3(e)–3(g)). Collectively, these data suggest that fraxetin had an inhibitory effect on the migration and invasion of glioma cells via downregulating MMP-2 and MMP-9.

### 3.4. Fraxetin Inhibits the Activation of JAK2/STAT3 Signaling in Glioma

**Figure 4:**
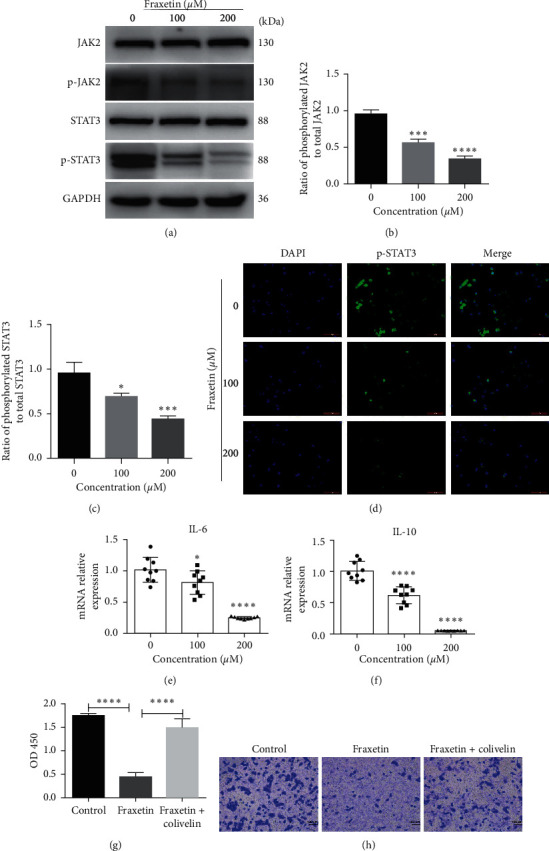
Fraxetin inhibits JAK2/STAT3 signaling in U251 cells. ((a), (b), and (c)) Western blot analysis of key proteins involved in JAK2/STAT3 signaling. Cells were treated with fraxetin (0 *μ*M, 100 *μ*M, and 200 *μ*M) for 24 h. (d) Immunofluorescence analysis of STAT3. Cells were treated with fraxetin (0 *μ*M, 100 *μ*M, and 200 *μ*M) for 24 h. ((e) and (f)) qRT-PCR analysis of IL-6 and IL-10. Cells were treated with fraxetin (0 *μ*M, 100 *μ*M, and 200 *μ*M) for 24 h. (g) CCK8 assay. Cells were treated with fraxetin (200 *μ*M) and Colivelin (10 *μ*M) together or fraxetin (200 *μ*M) alone for 24 h. (h) Transwell invasive assay. Cells were treated with fraxetin (200 *μ*M) and Colivelin (10 *μ*M) together or fraxetin (200 *μ*M) alone for 24 h. ^*∗*^*P* < 0.05; ^*∗∗∗*^*P* < 0.001; ^*∗∗∗∗*^*P* < 0.0001.

A previous study has shown that fraxetin inhibits the proliferation of lung cancer cells by inhibiting the activation of STAT3. Therefore, we hypothesized that fraxetin plays a protective role in U251 cells by targeting JAK2/STAT3 signaling. The results showed that there was no significant change in the expression of JAK2 and STAT3 proteins between the two groups, but the phosphorylation levels of JAK2 and STAT3 were obviously decreased after fraxetin treatment (Figures 4(a)–4(c)). Since the activation of JAK2/STAT3 signals is due to the nuclear expression of p-STAT3, an immunocytochemical staining was performed to detect the nuclear localization of p-STAT3. The results showed that fraxetin inhibits the nuclear expression of p-STAT3 in U251 cells (Figure 4(d)). Then, we measured IL-6 and IL-10, which were involved in immune response in tumor cells. As shown in Figures 4(e) and 4(f), fraxetin inhibited the expression of IL-6 and IL-10 mRNA. To further verify the inhibitory effect of fraxetin on the JAK2/STAT3 signaling pathway in glioma, we conducted experiments with the activator of JAK2/STAT3 pathway, Colivelin TFA. We found that Colivelin TFA can partially abolish the inhibitory effect of fraxetin on glioma (Figures 4(g) and 4(h)). Therefore, the JAK2/STAT3 signaling may be involved in fraxetin-mediated antitumor effects on glioma.

### 3.5. Fraxetin Inhibits Tumor Growth of Glioma Xenografts in Nude Mice

**Figure 5:**
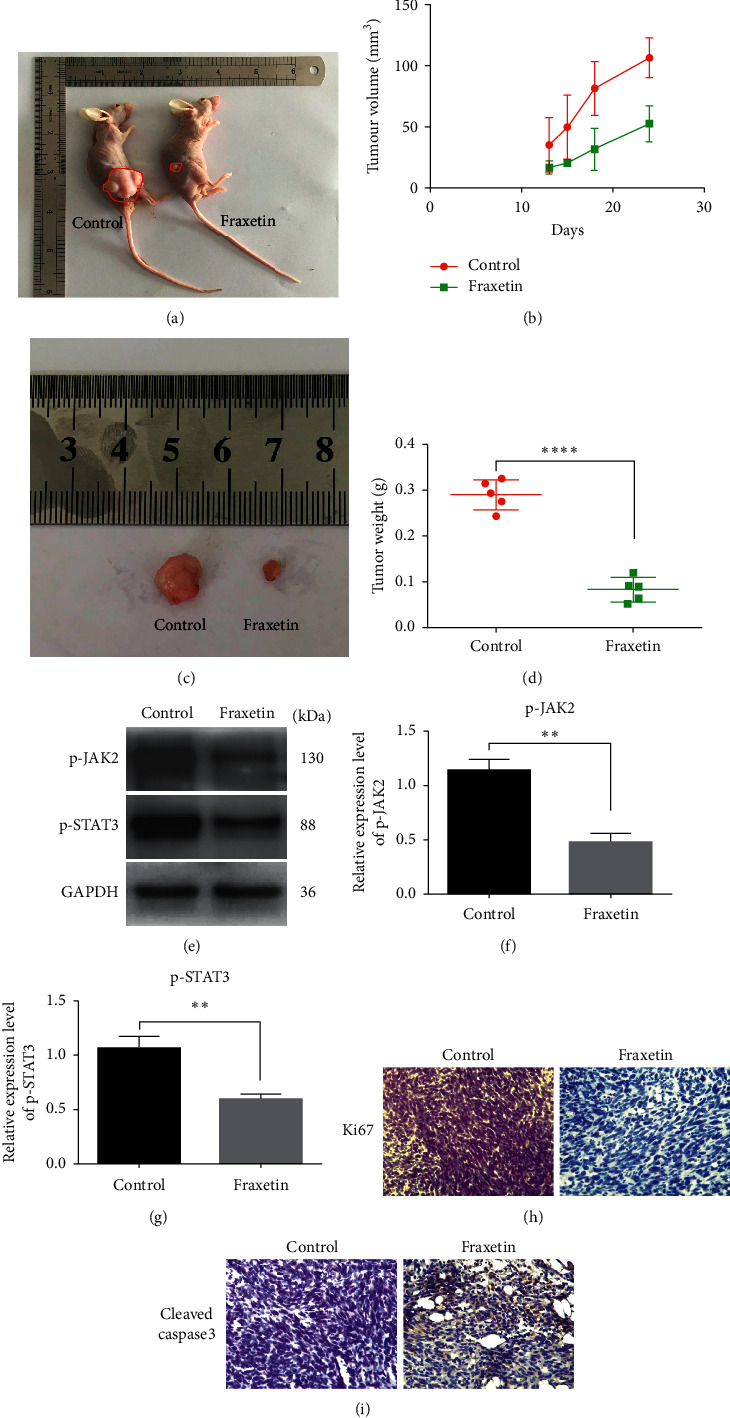
Fraxetin inhibits the growth of glioma xenografts in nude mice. (a) Picture of nude mice in control group and fraxetin-treatment group. (b) Tumor volume in control group and fraxetin-treatment group. (c) Picture of tumor in control group and fraxetin-treatment group. (d) Tumor weight of nude mice in control group and fraxetin-treatment group. ((e), (f), and (g)) Western blot analysis of key proteins involved in JAK2/STAT3 signaling in tumor. ((h) and (i)) histological analyses of Ki67 and cleaved caspase 3. ^*∗∗*^*P* < 0.01; ^*∗∗∗∗*^*P* < 0.0001.

To examine fraxetin's ability to inhibit proliferation, invasion, and migration of glioma cells *in vitro*, we performed *in vivo* experiments to determine whether a similar anticancer mechanism of fraxetin occurred in glioma xenografts of nude mice. We observed a significant decrease in tumor volume in fraxetin-treated group compared with the control group (Figures 5(a)–5(c)). Moreover, tumor weights in fraxetin-treated group were smaller than those in the control group (Figure 5(d)). Besides, the results of western blot showed that the expression levels of p-JAK2 and p-STAT3 proteins in mice treated with fraxetin were significantly decreased compared with the control group (Figures 5(e)–5(g)). In addition, histological analyses revealed that fraxetin inhibited the expression of Ki67, while cleaved caspase 3 was increased. Similar results were obtained from the *in vitro* study.

## 4. Discussion

The current treatment methods for glioma, including radiation and chemotherapy, are traditional. However, they are ineffective, particularly for advanced glioma [22]. The present advances in glioma chemotherapy suggest some effective drugs, such as vincristine, temozolomide, and lomustine. However, these drugs have side effects, due to targeting both normal cells and cancerous cells (nonspecific targeting) [23]. Temozolomide as an alkylating and methylating agent has been reported to be certainly less effective in patients with MGMT unmethylated tumor. Thus, better treatment options should be introduced urgently. Programmed cell death (PCD) is inhibited in cancer cells, and drugs promoting PCD are a potential specific chemotherapeutic treatment for cancers. Apoptosis is a type of PCD and is significant in chemotherapy [24]. Our results revealed that fraxetin, a plant-derived coumarin, could induce apoptosis of glioma cells (Figure 3(a)). The dose-dependent activation of PARP and caspase 3 further confirmed the conclusion (Figure 3(c)).

Extracellular matrix (ECM) and its substrates constitute the first barrier to tumor metastasis. Therefore, degradation of ECM is a critical link in tumor invasion and metastasis. As essential enzymes known to degrade extracellular matrix, MMPs play an important role in mediating tumor angiogenesis, metastasis, and invasion [25]. Type IV collagen has been reported to be the main component of the extracellular matrix and basement membrane, and fibrous collagen can be degraded by MMP-2 and MMP-9 after collagenase cleavage. The overexpression of MMP-2 and MMP-9 was associated with various tumor progressions, including rectal cancer, glioma, and papillary thyroid carcinoma. Therefore, MMP-2 and MMP-9 are considered as potential biomarkers for the process of tumor development and are important potential targets for antitumor therapy [26]. In this study, we found out that fraxetin inhibits the invasion and migration of glioma by downregulating the expression of MMP-2 and MMP-9 in a dose-dependent manner (Figure 3). These results suggest that fraxetin may be a potential treatment for glioma.

JAK2/STAT3 pathway is involved in various cellular processes, such as cellular differentiation, proliferation, migration, and apoptosis in mammals [7, 14, 27]. Thus, treatment targeting the JAK/STAT signaling pathway may be beneficial for patients with cancer. The inhibition of JAK2 signaling activates mitochondrial-dependent apoptotic pathways to induce cancer cell apoptosis [28]. STAT3, a downstream molecule of JAK2, is a member of the family of the signal transducer and activator of transcription (STAT), which is constitutively activated in most cancers, including glioma. The transcription factor STAT3 can promote tumor growth and angiogenesis, increase the invasion and migration of tumors, and inhibit immune responses [29–31]. Considering that the JAK2/STAT3 signaling pathway is overactivated in glioma cells [14], we speculate that fraxetin may play an antiglioma effect by inhibiting JAK2/STAT3 signaling. In this study, we found that fraxetin decreased the expression of phosphorylated JAK2 and phosphorylated STAT3 *in vivo* and *in vitro* (Figures 4(a) and 5(e)). Meanwhile, immunocytochemical staining showed that fraxetin inhibited the nuclear expression of p-STAT3 (Figure 4(d)). The data indicates that fraxetin inhibits glioma cancer cells through JAK2/STAT3 signaling.

In conclusion, we show that fraxetin, a naturally derived product, inhibits glioma proliferation, invasion, and migration and may be a valuable candidate for treatment of glioma. Mechanistically, fraxetin induces apoptosis through JAK2/STAT3 signaling pathway. This study provides a theoretical and experimental basis for the development of new methods and agents for the treatment of glioma.

## Data Availability

The datasets used and analyzed during the present study are available from the corresponding author on reasonable request.

## Abbreviations

DMEM: Dulbecco's modified Eagle medium
JAK2: Janus kinase 2
STAT3: Signal transducer and activator of transcription 3
RTCA: Real-time cell analysis
MMPs: Matrix metalloproteinases.

## Supplementary Materials

Supplementary Materials Figure S1: (A–B) CCK8 assay. BV2 and U87 cells were treated with fraxetin (0 *μ*M, 100 *μ*M, and 200 *μ*M) for 24 h. (C) Wound healing assay. U87 cells were treated with fraxetin (0 *μ*M, 100 *μ*M, and 200 *μ*M) for 24 h. (D) Transwell invasive assay. U87 cells were treated with fraxetin (0 *μ*M, 100 *μ*M, and 200 *μ*M) for 24 h. (E) CCK8 assay. U251 cells were treated with only DMSO. (F) Transwell invasive assay. U251 cells were treated with only DMSO. ^*∗∗∗∗*^*P* < 0.0001.
