# Supplementary figures and images for: Fraxetin Inhibits the Proliferation and Metastasis of Glioma Cells by Inactivating JAK2/STAT3 Signaling
Source: Evid Based Complement Alternat Med. 2021 Apr 16;2021:5540139. doi: 10.1155/2021/5540139 (PMC8075667; doi:10.1155/2021/5540139)

A

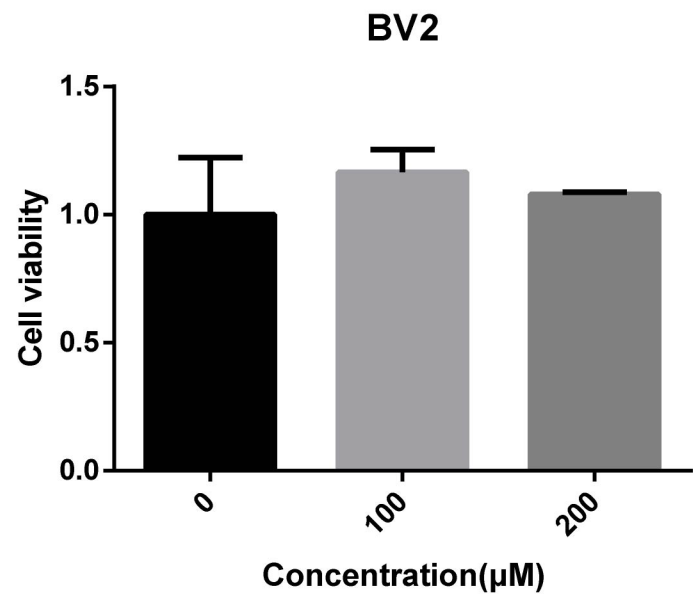

B

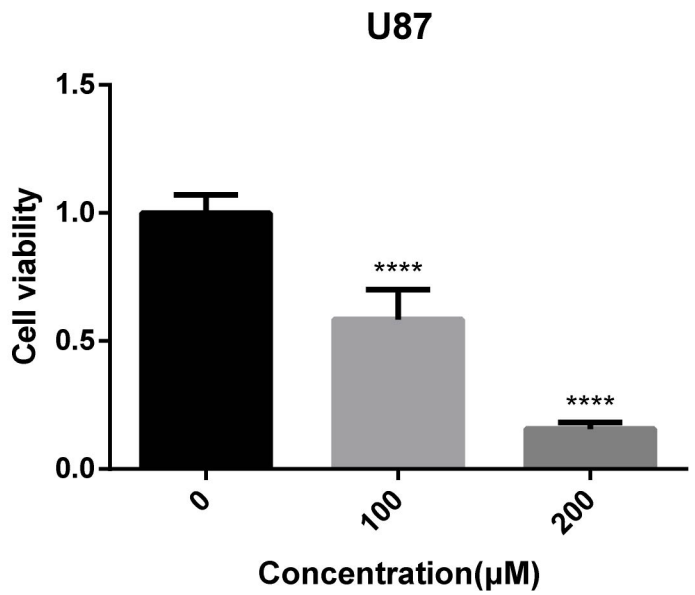

C

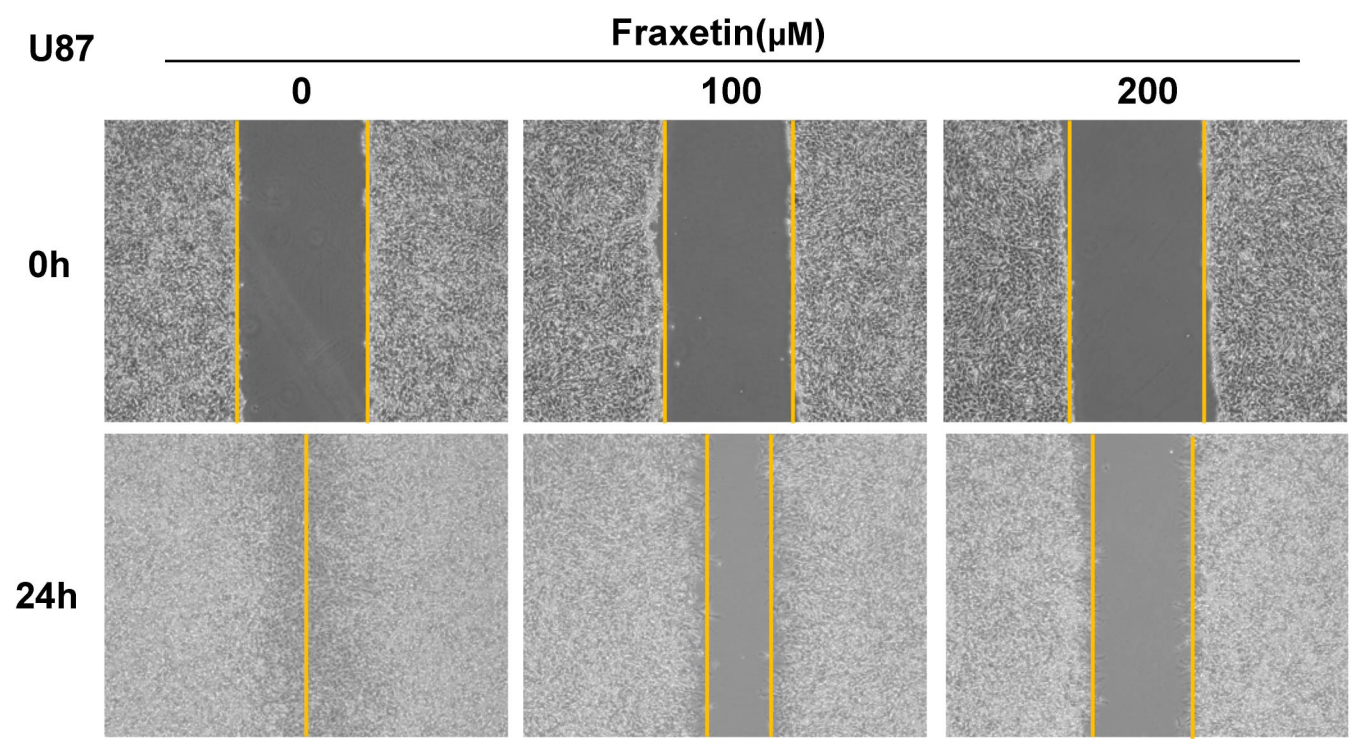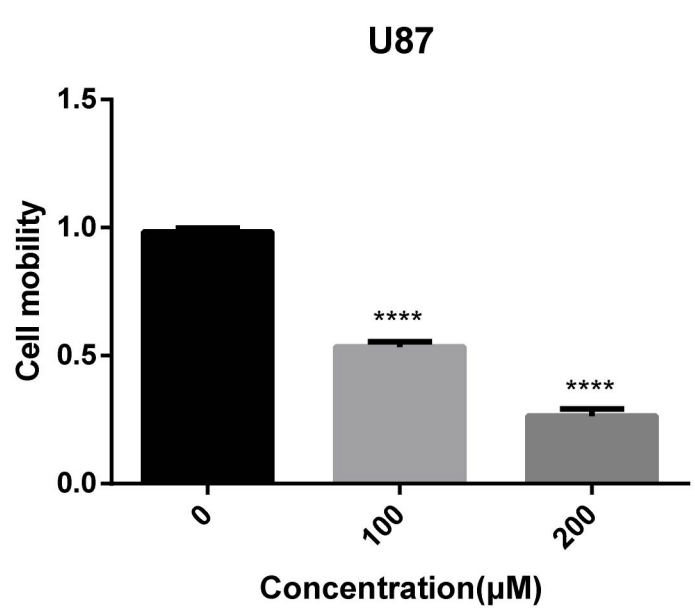

D

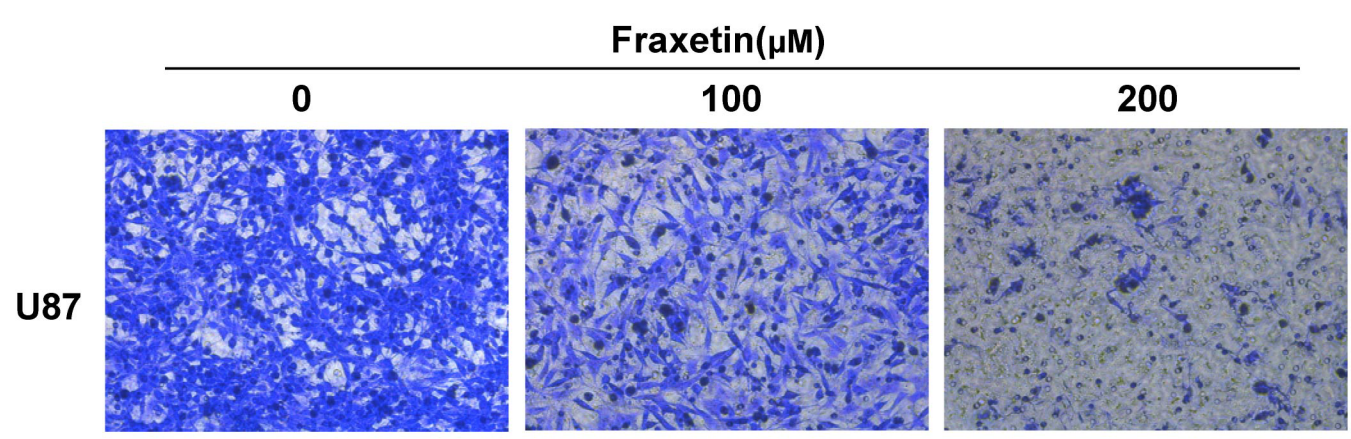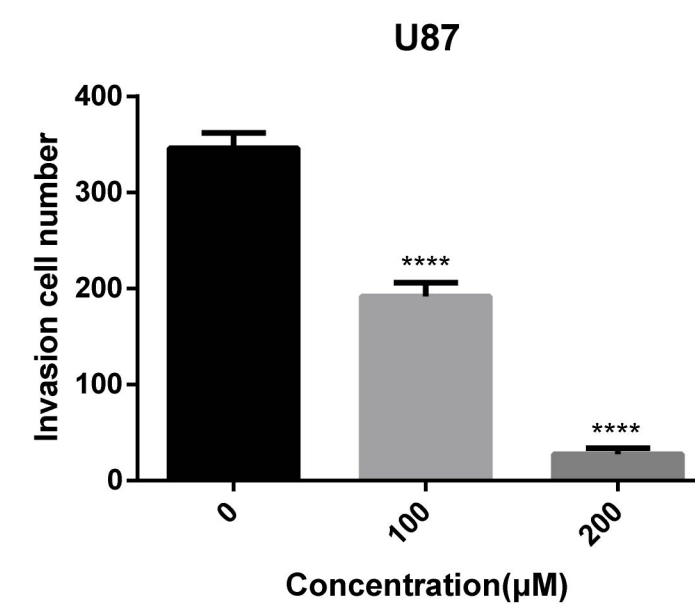

E

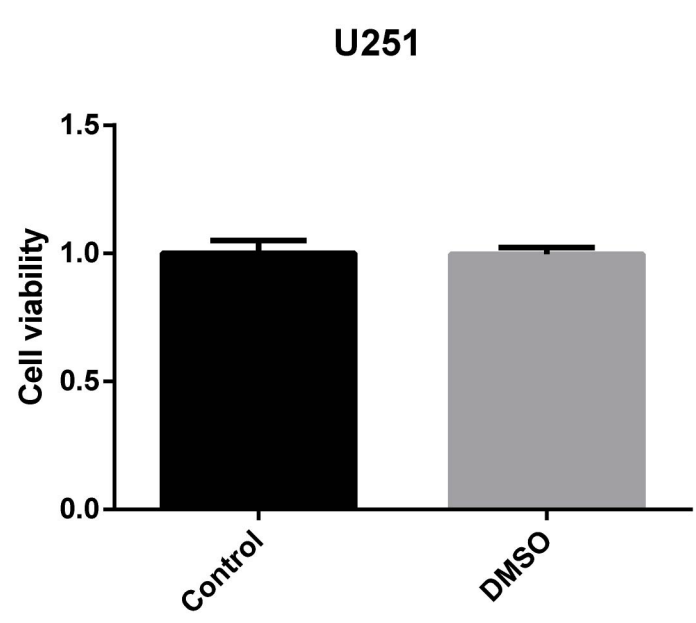

F

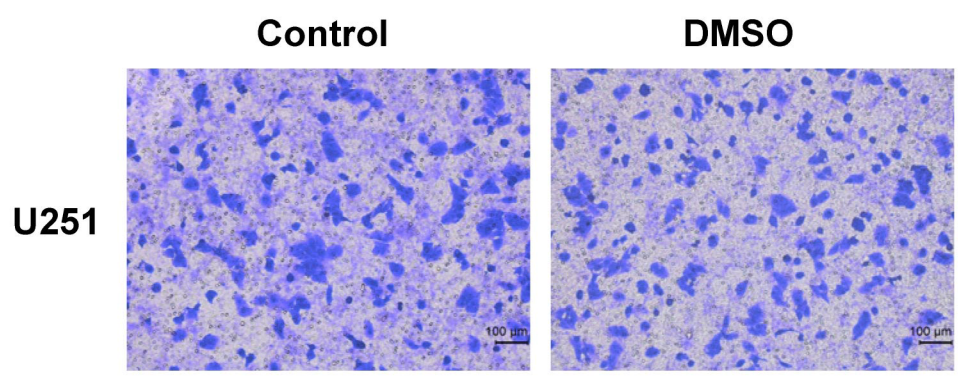

Supplement: Supplementary Materials — Figure S1: (A–B) CCK8 assay. BV2 and U87 cells were treated with fraxetin (0 μM, 100 μM, and 200 μM) for 24 h. (C) Wound healing assay. U87 cells were treated with fraxetin (0 μM, 100 μM, and 200 μM) for 24 h. (D) Transwell invasive assay. U87 cells were treated with fraxetin (0 μM, 100 μM, and 200 μM) for 24 h. (E) CCK8 assay. U251 cells were treated with only DMSO. (F) Transwell invasive assay. U251 cells were treated with only DMSO. ∗∗∗∗P < 0.0001. [file 5540139.f1.pdf]
